# School‐Based Weekly Iron–Folic Acid Supplementation and Its Effect on Mental Health Outcomes Among Adolescent Girls in Central Ethiopia: A Cluster‐Randomized Controlled Trial

**DOI:** 10.1155/jnme/9500345

**Published:** 2026-02-08

**Authors:** Shemsu Kedir, Dessalegn Tamiru, Beyene Wondafrash Ademe, Kalkidan Hassen Abate

**Affiliations:** ^1^ Department of Nutrition and Dietetics, Faculty of Public Health, Institute of Health, Jimma University, Jimma, Ethiopia, ju.edu.et; ^2^ Department of Public Health, College of Medicine and Health Sciences, Werabe University, Werabe, Ethiopia

**Keywords:** adolescent, common mental disorder, mental health, school, WIFAS

## Abstract

**Background:**

Common mental disorders (CMDs) are increasingly recognized as a major public health challenge worldwide, with adolescent girls bearing a disproportionate burden. Moreover, iron deficiency (ID) and iron deficiency anemia (IDA) remain prevalent nutritional problems among adolescents. Observational studies have highlighted that the prevalence of CMD is higher among anemic individuals compared to their non‐anemic counterparts. This study hypothesizes that preventing anemia through weekly iron and folic acid supplementation (WIFAS) among adolescent girls may have a positive effect on CMDs. However, the potential effect of WIFAS on CMDs remains unexplored. This critical evidence gap highlights the need for targeted research. Therefore, this study aimed to assess the effect of WIFAS on CMD among adolescent girls in the Central Ethiopia Region.

**Method:**

This study employed a school‐based, parallel‐cluster randomized controlled trial (RCT) design, involving 306 adolescent girls across 12 clusters in the Silti and Kibet Districts of the Central Ethiopia Region. Participants were proportionally allocated, with 153 girls assigned to the intervention group (six clusters in Silti District) and 153 to the control group (six clusters in Kibet District). The outcome variable, CMD score, was treated as a continuous measure and assessed using the World Health Organization’s (WHO) Self‐Reporting Questionnaire (SRQ‐20). Data were collected at baseline and endline, with the latter conducted 6 months after the completion of the WIFAS. Linear mixed‐effects models (LMEMs) were used to examine the association between WIFAS and CMD.

**Result:**

The findings of this study indicated that adolescents who received WIFAS had significantly lower CMD scores compared to those who did not (*β* = −1.12; 95% CI: −1.68, −0.55). The unadjusted mean difference was −0.88 (Model 1: *β* = −0.88; 95% CI: −1.47, −0.29). This association remained significant after adjusting for baseline CMD score (Model 2: *β* = −1.01; 95% CI: −1.58, −0.45), parental sociodemographic characteristics (Model 3: *β* = −1.12; 95% CI: −1.68, −0.55), and in the fully adjusted model (Model 4: *β* = −1.12; 95% CI: −1.68, −0.55).

**Conclusion:**

The findings of this study revealed that WIFAS significantly reduced CMD scores among adolescent girls. This suggests that WIFAS may offer mental health benefits in addition to its known role in preventing anemia and ID. Hence, integrating WIFAS into school‐based health programs should be prioritized as a potential strategy to improve mental health outcomes among adolescent girls.

**Trial Registration:** ClinicalTrials.gov identifier: PACTR202309541331083

## 1. Introduction

Common mental disorders (CMDs) are defined by symptoms of sleeplessness, exhaustion, irritability, poor memory, difficulty concentrating, and somatic complaints [1, 2]. However, according to the International Classification of Diseases‐10 (ICD‐10), CMD may include “neurotic, stress‐related, somatoform disorders” and “mood disorders” that usually manifest with the occurrence of a combination of nonspecific anxiety, depressive, and somatic symptoms [3]. In 2019, mental disorders affected approximately one in eight individuals globally, representing a substantial epidemiological and public health concern [4]. Additionally, evidence suggests that nearly half of the population (about 44%) will encounter at least one mental disorder episode in their lifetime [5]. According to the Global Burden of Disease (GBD) 2021, the total burden of mental disorders continues to rise, with notable variations across genders, regions, and disorder subtypes. Greater attention is needed to address the disproportionate disease burden among females [6].

The Lancet journal’s GBD study revealed that 16% of adolescent age groups (10–19 years) are affected by mental health conditions [7]. Adolescent girls in Ethiopia face a dual burden of nutritional deficit and psychological distress. The prevalence of CMD among adolescents in Ethiopia ranges from 20.7% to 47% [8–12]. In middle adolescence, particularly around the age of 14, the majority of CMD cases remain undiagnosed, and most cases tend to emerge during this period [13]. Similarly, anemia is also widespread among adolescent girls in Ethiopia. A recent systematic review and meta‐analysis found a pooled prevalence of about 23.0% (95% CI: 17.2%–28.8%) [14]. In specific localities, prevalence rates are even higher: for instance, 29% in three districts in Ethiopia [15], 26.7% in Jimma Town [16], 29% in Silti District [17], and 29.4% in Haramaya Town [18]. In addition, there is emerging local evidence linking anemia (or low hemoglobin) with CMDs. For example, a structural equation model study in Central Ethiopia reported that hemoglobin level negatively correlates with CMD symptoms and that morbidity symptoms and animal‐source food intake (which influence hemoglobin) also indirectly affect CMDs [19].

Due to multifactorial causes, CMDs are not limited to biological, social, and economic factors [20–23]. Adolescence is a span of rapid linear growth and significant neurodevelopmental changes [24–26]. To attain this development and growth in the adolescent age group, nutritional requirements are heightened relative to childhood [27]. CMD typically arises during adolescence, a period marked by increased nutritional demands [28]. In particular, there has been a growing focus on investigating iron metabolism and its pivotal role in the context of mental disorders [29]. Norepinephrine has been linked to iron metabolism in the brain, which can affect the neuroplasticity and function of prefrontal neurons and the hippocampus. Iron deficiency anemia (IDA) and iron deficiency (ID) have been significantly associated with alteration of monoamine neurotransmitters and abnormal white matter myelination and are likely related to mental health problems [30, 31]. In addition, a high prevalence (up to 30%) of IDA was observed in children, which potentially affects their communication and behavior [32]. Moreover, a nationwide database analysis study indicates that IDA subjects had an increased risk of psychiatric disorders, regardless of other confounders. In IDA patients, iron supplementation was associated with a decreased risk of psychiatric disorders [29]. Research in unmedicated adolescent females found that lower ferritin levels were linked to more severe depression, anxiety, and smaller brain region volumes [33]. Furthermore, a Japanese survey also showed that self‐reported IDA was associated with higher psychological distress and depression in both sexes [34]. Because iron plays a key role in the metabolism of monoamines in the brain, ID leads to symptoms such as apathy, drowsiness, irritability, and lack of attention that occur due to impaired monoamine oxidase activity [35].

To mitigate this interrelated problem, nutritional intervention is crucial in many low‐ and middle‐income countries (LMICs), where micronutrient deficiencies, such as ID, are common and supplementation may serve as a cost‐effective public health strategy [36]. In brain development, iron is responsible for myelination of white matter [37, 38] and the development and function of the various neurotransmitter systems, including the dopamine, norepinephrine, and serotonin systems [39, 40]. Schools serve as a strategic platform for delivering integrated health and nutrition interventions to adolescents, including micronutrient supplementation, infection control, and health education [41].

Despite the evidence for high anemia prevalence and clear theoretical mechanisms (impaired oxygen transport, neurotransmitter synthesis) by which ID could influence mental health, little is known about the effect of weekly iron and folic acid supplementation (WIFAS) on mental health outcomes in this context. While WIFAS is recommended for adolescent girls in many LMICs, including Ethiopia, program coverage, adherence, and local evidence on effects beyond anemia (i.e., on CMDs) are scarce or not well documented in published literature. In the current study area, there is a high burden of anemia among adolescent girls, and there is a significant proportion of adolescent girls who face CMD, often linked to anemia [17, 19]. Moreover, a recent systematic review and meta‐analysis [42] found no eligible studies evaluating the effects of WIFAS on mental health outcomes, revealing a critical gap in evidence. This lack of data underscores the need for targeted research to determine whether WIFAS could play a role in preventing or mitigating CMDs among adolescents, besides preventing anemia. Thus, this study aimed to examine the effect of WIFAS on CMD among adolescent girls in the Central Ethiopia Region.

## 2. Methods

### 2.1. Study Setting

The study was conducted in the Silti and Kibet District, Silte Zone, Central Ethiopia Region, which is located about 144 km away from the capital city of Ethiopia, Addis Ababa. These districts are predominantly dependent on cereal‐based agriculture, practice mixed crop–livestock production, and live in permanent settlements. According to the 2022 report, the total estimated population of Kibet and Silti District was 32,755 and 151,573, respectively. In 2023, student enrollment data showed that these districts had 21,725 school‐attending adolescents, of whom 10,669 were girls. Among them, 7721 were enrolled in primary schools, and 2948 were attending secondary schools. Across the Silti and Kibet Districts, 37 public schools were identified, where 28 were located in rural areas and 9 in urban settings. In total, the districts comprise 30 primary schools and 7 secondary schools. Baseline data were collected between October 2 and 20, 2023. The intervention was implemented over six months, running from October 31, 2023, to April 30, 2024. Endline data were gathered from May 7 to 31, 2024.

### 2.2. Study Design, Sample Size, and Study Population

A school‐based clustered randomized controlled trial (RCT) design with two arms, an intervention group and a control group, was employed. The sample size was calculated separately for each primary outcome (CMD score, school performance, and growth indicators), and the largest value was selected to ensure adequate statistical power across all study objectives. This required sample size was calculated using G‐power 3.1.9.4, based on the following parameters, including a mean weight change difference of 2.4 [43], one tail, a power of 80%, *α* (level of significance) 0.05, and an allocation ratio of 1. Design effect (DE) of 2.2 was applied, calculated as DE 1 + (*m* − 1) ∗ ICC, where *m* = 25 and ICC = 0.05. Additionally, a 20% loss to follow‐up was considered, resulting in a final sample size of 306 participants. School adolescent girls aged 10–19 years were randomly selected for inclusion in the study. However, those who planned to leave school within the next 6 months, had a diagnosis of anemia, or were regularly taking prescribed or habitual micronutrient supplements were excluded.

### 2.3. Recruitment and Randomization

**FIGURE 1:**
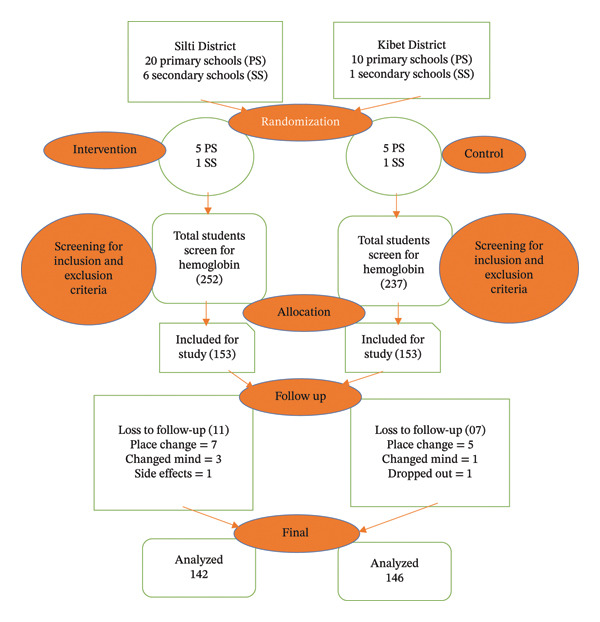
Flow of the study participants through the trial according to the criteria recommended in the CONSORT guideline.

Schools were treated as clusters, with six clusters (five primary and one secondary) randomly selected for the intervention arm from 20 primary and six secondary schools in the Silti District. Likewise, six clusters (five primary and one secondary) were chosen for the control arm from 10 primary and one secondary school in the Kibet District. Official school records indicated that 3480 adolescent girls were enrolled in primary schools and 898 in secondary schools. Using these data, a sampling frame was developed to select a total of 306 participants, 153 in each arm, through proportional allocation. The final sample was generated using ENA software, considering the selection of 25 to 26 students from each school based on data from school registries. Study participants were screened and enrolled by nurses, clusters were randomly assigned, and a school‐based survey was conducted, during which eligible adolescent girls were screened for anemia via hemoglobin measurement (Figure 1).

### 2.4. Intervention Allocation and Follow‐Up Procedures

The intervention group received school‐based WIFAS supplementation for 6 months, while the control group received only the usual healthcare services. The intervention involved administering one tablet per week, each containing 60 mg of elemental iron and 2800 mcg of folic acid. One teacher from each selected school was assigned to oversee the supplementation process, and two health officers acted as supervisors to ensure proper implementation every week. Teachers and health officers monitored the participants weekly during supplementation to assess their general health and any side effects, such as black stools, nausea, constipation, abdominal cramps, and vomiting. Tablets were administered about 1 hour after breakfast. If vomiting occurred within 30 min of ingestion, a replacement dose was given.

### 2.5. Data Collection Measurements

The structured questionnaire was translated into Amharic and Siltigna, the main local languages, and field‐tested before use in accordance with recommended guidelines. The mental health status, specifically the presence of CMDs, was assessed by using a standardized Self‐Reporting Questionnaire (SRQ‐20), a 20‐item retrospective instrument developed by the World Health Organization (WHO) [44, 45]. This comprises dichotomous (yes/no) responses that evaluate psychiatric symptomatology. The questionnaire includes both somatic symptoms (e.g., headaches, loss of appetite, fatigue) and psychological symptoms (e.g., feelings of unhappiness, nervousness, and worthlessness). Each participant received a mental health score ranging from 0 to 20, with higher scores indicating greater symptom severity. This variable was treated as a continuous measure in the analysis.

The Household Food Insecurity Access Scale (HFIAS) questionnaire, which includes a list of 9 specific questions about worry, availability, and accessibility to foods for the household during the previous 30 days, was used to collect household food insecurity. Response options for each question were based on a Likert scale: *never*, *rarely*, *sometimes*, and *often*, coded as 0, 1, 2, and 3, respectively. The food insecurity score was calculated by summing responses across all items. Households with a total score between 0 and 2 were classified as food secure, while those with scores between 2 and 27 were considered food insecure [46, 47].

Dietary data were collected using an adapted food frequency questionnaire containing 28 food items, originally developed in the nearby town of Butajira [48]. After data collection, the items were grouped into nine food categories based on the Individual Dietary Diversity Score (IDDS) framework provided by the FAO. Food frequency questionnaires are particularly useful for ranking individuals according to their intake of specific food items, allowing for comparisons between those with high and low consumption levels [49]. An adolescent who consumed at least one item from each of the nine food groups during the week would receive the maximum Dietary Diversity Score (DDS) of 9, while those who did not consume any items from the food groups would receive a score of 0. Then, the minimum dietary diversity score of five or more out of the nine groups of foods was considered adequate.

The household wealth index was constructed using principal component analysis (PCA), based on 38 variables such as ownership of household assets, agricultural land, livestock, access to water, type of dwelling, availability of a latrine, and bank account ownership [50, 51]. Nondummy variables were binary coded (1 = *present*, 0 = *absent*) in accordance with WHO/UNICEF guidelines. Based on the PCA results, households were stratified into wealth quintiles and categorized as poor, medium, or rich for further analysis.

### 2.6. Data Management and Analysis

Data were entered using Kobo Toolbox and exported to Stata Version 14 for analysis. Sociodemographic differences at baseline between the two groups were evaluated using the chi‐square test. Due to the data’s violation of the assumption of normal distribution, characterized by a right‐skewed shape, nonparametric methods were applied. The Wilcoxon Signed‐Rank Test was used to determine significant intra‐group change (pre‐to‐post intervention), while the Mann–Whitney *U*‐test was applied to assess significant inter‐group differences. The distribution issues likely stem from the nature of the scale, which is bounded by a minimum value of 0 and a maximum of 20. The outcome variable, CMD, was treated as a continuous measure, ranging from 0 to 20. The effect of the intervention on changes in the mental health status of adolescents over time was estimated using a linear mixed‐effects model (LMEM), which allowed us to explain how the results were correlated. The maximum likelihood test was used to help us choose the best statistical model. The model that displayed the highest likelihood test was selected.

Variables with *p* values less than 0.2 in the bivariate analysis were considered for the multivariate linear mixed model, with participant‐level variability accounted for by including individuals as random effects in the model. The intracluster correlation coefficient (ICC) of the final model was 0.05, indicating that 5% of the total variance in the outcome variable is attributable to differences between clusters (e.g., schools), while the remaining 95% of the variance is due to individual‐level differences within clusters.

### 2.7. Ethics Consideration

Ethical approval for the study was obtained from the Institutional Review Board (IRB) of Jimma University (Ref. No. JUIH/IRB/583/23). A support letter was also secured from the Silti and Kibet Education Offices to facilitate access to the selected schools under their jurisdiction. The purpose and procedures of the study were clearly explained to each school administration before data collection. After obtaining school approval, written parental consent was collected through consent letters sent with the students, and verbal assent was obtained from each adolescent participant. Each study participant received a thorough description of the study’s objective, protocol, and duration, as well as the potential risks and benefits, before providing informed consent. Coding and aggregated reporting were employed to remove names and other personal identifiers, ensuring participant anonymity, privacy, and confidentiality throughout the study.

## 3. Results

Of the 306 study participants, 288 (94.1%) were included in the final analysis. In terms of dietary diversity, 34.5% of participants in the intervention group and 34.2% in the control group consumed fewer than five food groups. More than two‐thirds (71%) of participants from both groups resided in rural areas. Additionally, the majority (66%) of participants were in the middle age category, ranging from 14 to 16 years. At baseline, there was no significant difference in socio‐demographic characteristics between the intervention and control groups (*p* > 0.05) (Table 1).

**TABLE 1:** Baseline sociodemographic characteristics of adolescent girls in Silti and Kibet Districts, Central Ethiopia Region, 2024.

Variables	Category	Intervention group *n* (%) or mean ± SD	Control group *n* (%) or mean ± SD	*p* value
Wealth index	Poor	51 (35.9)	49 (33.5)	0.61
	Middle	47 (33.1)	62 (42.5)	
	Rich	44 (31)	35 (24)	

IDDS	≥ 5	93 (65.5)	96 (65.8)	0.96
	< 5	49 (34.5)	50 (34.2)	

HFIAS	Secure	81 (57)	99 (67.8)	0.06
	Insecure	61 (43)	47 (32.2)	

Residence	Rural	101 (71.1)	102 (69.9)	0.82
	Urban	41 (28.9)	44 (30.1)	

Student age in years	Early adolescent (10–13)	28 (19.7)	21 (14.4)	0.33
	Middle adolescent (14–16)	89 (62.7)	97 (66.4)	
	Late adolescent (17–19)	25 (17.6)	28 (19.2)	

At baseline, a Mann–Whitney *U*‐test demonstrated no significant difference was observed between intervention and control groups (*p* = 0.072). Similarly, at endline, CMD scores were significantly lower in the intervention group compared with controls (*U* = 8902.0, *Z* = −2.21, *p* = 0.027). A Wilcoxon Signed‐Rank test showed a significant reduction in CMD scores within the intervention group (*Z* = −4.52, *p* < 0.001) but not in the control group (*Z* = −0.27, *p* = 0.791) (see Table 2).

**TABLE 2:** Changes in CMD scores (median, interquartile range [IQR] among adolescent girls, showing within‐ and between‐group comparisons by WIFAS intervention status in the Silti and Kibet Districts, Central Ethiopia Region, 2024.

Outcome variable	Period	Intervention group	Control group	Between‐group comparison
		Median ± (IQR)	Median ± (IQR)	*Z* and *p* value
CMD scores	Baseline	2 (0–6)	0 (0–4)	*Z* = −1.80, *p* = 0.072
	Endline	0 (0–2)	1 (0–5)	*Z* = −2.21, *p* = 0.027

Within‐group comparison	*Z* and *p* value	*Z* = −4.52, *p* < 0.001	*Z* = −0.27, *p* = 0.791	

In the linear mixed‐effects regression analysis, adolescents who consumed WIFAS had a significantly reduced CMD compared to those who did not, with an unadjusted mean difference of 0.88 (Model 1: *β* = −0.88, 95% CI: −1.47, −0.29). This negative association persisted after sequential adjustments for baseline CMD score (Model 2: *β* = −1.01, 95% CI: −1.58, −0.45), parental sociodemographics (Model 3: *β* = −1.11, 95% CI: −1.58, −0.55), and in the final adjusted model (Model 4, fully adjusted: *β* = −1.12, 95% CI: −1.68, −0.55) (Table 3).

**TABLE 3:** Linear mixed‐effects model assessing the effect of the WIFAS intervention on endline CMD score among adolescent girls in the Silti and Kibet Districts, Central Ethiopia Region, 2024.

Model	LMM		Intercepts estimate		Random effect estimate	Covariates	*p* value	Maximum likelihood ratio test
	*β* ± SE	95% CI	Mean ± SE	95% CI	Variance ± SE			
1	−0.88 (0.29)	−1.47, −0.29	2.36 (0.21)	1.95, 2.77	2.5 (0.105)	Unadjusted	0.003	−677
2	−1.01 (0.29)	−1.58, −0.45	1.9 (0.22)	1.46, 2.35	2.45 (0.102)	Adjusted for baseline mental health score	0.0001	−666.9
3	−1.11 (0.28)	−1.68, −0.55	1.9 (0.34)	1.22, 2.57	2.39 (0.09)	Adjusted for the education status of the mother, wealth index, and HFIAS	0.0001	−660
4	−1.12 (0.28)	−1.68, −0.55	2 (0.48)	1, 2.9	2.37 (0.09)	Adjusted for the student’s DDS, residence, and age category	0.0001	−658

*Note:* CI, confidence interval; DDS, dietary diversity score; HFIAS, Household Food Insecurity Access Scale; LMM, linear mixed effect model; SE, standard error; and *β*‐coefficient, mean effect size.

## 4. Discussion

This trial aimed to assess the effect of school‐based WIFAS (a 6‐month intervention) on CMD scores among adolescent girls. Model 4, the fully adjusted LMEM, was designated as the preferred model based on the maximization of the Log‐Likelihood value, indicating the best overall statistical fit to the data. Results from this model confirmed that the WIFAS intervention was a significant determinant of the endline mental health outcome, demonstrating a 1.12‐point reduction in mean CMD scores (*β* = −1.12, *p* < 0.0001) of the intervention group compared to the control group. This effect proved to be robust after adjusting for the full set of baselines, individual‐level (e.g., age, residence, DDS), and household‐level (e.g., wealth index, HFIAS, mother’s education status) covariates.

The baseline prevalence of anemia in this study was 29%, while the prevalence of CMDs was 22.3%. Furthermore, CMDs were more prevalent among anemic individuals compared to their non‐anemic counterparts. Additionally, hemoglobin level (anemia) was identified as a significant mediator in the relationship between the number of morbidity episodes and CMDs [17, 19]. These findings suggest that improving hemoglobin levels through anemia prevention strategies, such as iron supplementation, may help reduce the burden of CMDs among adolescent girls. Consistent with this, WIFAS supplementation in this study had a positive effect on hemoglobin concentration and serum ferritin levels​ [52], indicating its potential role in addressing both anemia and related mental health outcomes.

Iron supplementation may alleviate psychological symptoms associated with hypoferritinemia in children and adolescents, including anxiety, depression, low energy, irritability, and difficulty concentrating [53]. This might be because iron is essential for the synthesis of neurotransmitters, which play critical roles in mood regulation. Hence, deficiencies in iron can disrupt these pathways, potentially leading to mood disorders. Moreover, ID may affect brain structures involved in emotion processing, such as the basal ganglia.

A placebo‐controlled trial in young teen mothers also showed a positive response to iron therapy in cognitive performance variables, but variables of emotional status were not thoroughly studied [54]. Weekly preconception micronutrient supplements containing iron may have benefitted women who were at risk for depression [55]. Similarly, supplementation with FA‐containing micronutrients may be equally efficacious in improving symptoms of depression when provided daily or weekly [56]. The findings of this study show a clearer beneficial effect of WIFAS on adolescent mental health, which might be due to adolescents being more vulnerable to ID’s neurological effects than adults and may be due to the supplementation that contains folic acid.

Similarly, the findings of a systematic review and meta‐analysis indicated that ID correlated with worse mental health scores and greater fatigue at baseline [57]. Moreover, IDA patients receiving iron supplementation also had a lower risk of sleep disorders [29]. Research involving unmedicated adolescent females found that lower ferritin levels were associated with more severe depressive and anxiety symptoms. Additionally, reduced ferritin correlated with smaller volumes in certain brain regions, suggesting a potential impact on brain development [33].

A key strength of this study is its school‐based design along with cluster RCT, which enabled access to a large population of adolescent girls. It is also the first study to assess the effects of WIFAS on CMDs among adolescents aged 10–19 over 6 months, particularly in Ethiopia. Additionally, hemoglobin levels were measured to identify the eligibility criteria before the intervention. However, this study has some limitations. Mental health was assessed only at baseline and endline using a self‐reported WHO questionnaire, which may not capture fluctuations over time. Although adjustments were made for wealth index, family size, residence, age, grade, and dietary factors such as HFIAS and IDDS, other unmeasured variables (residual confounding) (e.g., genetic factors, infection status, bioavailability of nutrients, or recall bias in dietary reporting) might still have influenced the observed associations. Similarly, even though baseline characteristics were compared and found to be generally similar, residual differences between districts cannot be ruled out and may have influenced the observed outcomes due to district‐level cluster randomization. Furthermore, the random effect variance of 2.37 remains high and largely unexplained, suggesting that important contextual factors (e.g., school climate, teacher–student relationships, peer environment, or access to health services) are driving differences in student mental health across the districts. Future research could explore the long‐term effects of WIFAS using extended supplementation periods and alternative mental health assessment tools, along with addressing school‐level factors and other residual confounding variables.

## 5. Implication of the Study

### 5.1. Policy Implications

The consistent association between WIFAS and reduced CMD symptoms suggests that iron–folic acid supplementation may have mental health benefits beyond its established role in preventing anemia. This finding supports integrating WIFAS into national adolescent health policies not only as a nutrition intervention but also as a complementary mental health promotion strategy. Ministries of Health and Education should consider strengthening school‐based supplementation programs and including mental health indicators in routine monitoring systems. Furthermore, the results justify policy‐level investment in adolescent supplementation programs, particularly in regions with high anemia prevalence and growing mental health concerns.

### 5.2. Program‐Level Implications

Programs implementing WIFAS may observe secondary benefits in emotional well‐being and reduction in CMD symptoms among adolescents. Therefore, supplementation programs could be strategically linked with school mental health initiatives and psychosocial support platforms.

## 6. Conclusion

This study demonstrated that WIFAS significantly reduced CMD scores among adolescent girls, indicating a potential mental health benefit beyond its established role in preventing anemia and ID. The consistent negative association observed across multiple regression models highlights the possible psychological advantages of WIFAS in this population. Given the dual burden of anemia and mental health issues among adolescent girls in low‐resource settings, integrating WIFAS into existing school‐based health and nutrition programs should be considered a priority. Additionally, further longitudinal research is warranted to investigate the long‐term mental health impacts of WIFAS and to elucidate the biological mechanisms (causal pathway) underlying these effects.

## Author Contributions

Conceptualization and data curation: Shemsu Kedir, Beyene Wondafrash Ademe, and Kalkidan Hassen Abate; methodology and supervision: Shemsu Kedir, Dessalegn Tamiru, Beyene Wondafrash Ademe, and Kalkidan Hassen Abate; formal analysis: Shemsu Kedir, Dessalegn Tamiru, Beyene Wondafrash Ademe, and Kalkidan Hassen Abate; validation: Shemsu Kedir, Dessalegn Tamiru, Beyene Wondafrash Ademe, and Kalkidan Hassen Abate; writing–original draft: Shemsu Kedir; writing–review and editing: Shemsu Kedir, Dessalegn Tamiru, Beyene Wondafrash Ademe, and Kalkidan Hassen Abate.

## Funding

The study was funded by Jimma University and Werabe University with the reference numbers JUIH/IRB/583/2023 and WRU/RP&CS‐VP/2023, respectively.

## Disclosure

All authors read and approved the final manuscript. The funders had no role in study design, data collection, and analysis, the decision to publish, or preparation of the manuscript.

## Conflicts of Interest

The authors declare no conflicts of interest.

## Data Availability

The data used and/or analyzed during the current study are available from the corresponding author based on a reasonable request.
